# Lung Ultrasound in Bronchopulmonary Dysplasia: Diagnostic Tool, Prognostic Marker or Monitoring Strategy?

**DOI:** 10.3390/children13081103

**Published:** 2026-08-18

**Authors:** Ilaria Bucci, Dorina Hoxha, Chiara Rosolia Capasso, Sabrina Di Pillo, Francesco Chiarelli, Marina Attanasi, Paola Di Filippo

**Affiliations:** Pediatric Allergy and Pulmonology Unit, Department of Pediatrics, University of Chieti-Pescara, Via dei Vestini n°5, 66100 Chieti, Italy; ilaria.bucci@studenti.unich.it (I.B.); dorina.hoxha@studenti.unich.it (D.H.); chiara.rosolia.capasso@studenti.unich.it (C.R.C.); sabrina.dipillo@asl2abruzzo.it (S.D.P.); chiarelli@unich.it (F.C.); marina.attanasi@unich.it (M.A.)

**Keywords:** bronchopulmonary dysplasia, lung ultrasound, prematurity, longitudinal monitoring, artificial intelligence

## Abstract

Bronchopulmonary dysplasia (BPD) remains one of the leading chronic respiratory complications of extreme prematurity despite major advances in neonatal intensive care. Current diagnostic definitions are primarily based on respiratory support requirements and provide limited information on the biological heterogeneity and longitudinal evolution of lung injury. Lung ultrasound (LUS) is progressively reshaping the clinical approach to BPD by enabling radiation-free, bedside, and repeatable assessment of peripheral lung abnormalities throughout the neonatal course. This narrative review examines the current role of LUS in BPD, integrating its pathophysiological basis with the available evidence and distinguishing explicitly between its diagnostic, prognostic, monitoring, and treatment-guiding applications, which are supported by markedly different levels of evidence. We discuss how LUS findings reflect the major components of BPD pathophysiology, compare LUS with conventional imaging modalities, and summarize the scanning protocols, semiquantitative scoring systems, examination schedules, and patient populations that have been evaluated to date. We also review the emerging contribution of artificial intelligence to automated image analysis, standardized image acquisition, and personalized risk prediction. Most of the available evidence is prognostic rather than diagnostic: early LUS scores predict BPD subsequently defined at 36 weeks’ postmenstrual age, whereas LUS has not been validated as a diagnostic test for established BPD, and reported cutoffs remain study- and protocol-specific rather than universally applicable. Randomized evidence is confined to LUS-guided surfactant administration, where the demonstrated benefits concern the timing of treatment and the need for invasive ventilation; no trial has yet shown that LUS-guided management reduces the incidence of BPD. Current evidence supports LUS as a complementary imaging modality that extends beyond the assessment of acute neonatal respiratory disease and enables longitudinal bedside monitoring of peripheral lung aeration. Although further multicenter prospective studies are required to harmonize protocols and validate LUS-guided management strategies, the integration of standardized LUS assessment with emerging artificial intelligence technologies has the potential to establish LUS as a key component of precision respiratory care for infants at risk of BPD.

## 1. Introduction

Bronchopulmonary dysplasia (BPD) is the most common chronic respiratory complication of extreme prematurity and remains a major cause of neonatal morbidity despite substantial advances in perinatal and neonatal care. Originally described by Northway and colleagues in 1967, the disease has evolved considerably over the past five decades alongside improvements in neonatal intensive care and the increasing survival of extremely preterm infants [1]. While early descriptions relied heavily on radiographic abnormalities, contemporary definitions are primarily based on the need for supplemental oxygen or respiratory support at specific postnatal ages [2,3].

BPD is now recognized as a developmental lung disease resulting from the interaction between interrupted lung maturation and multiple antenatal and postnatal insults, including inflammation, oxygen toxicity, infection, nutritional deficiency, and ventilator-induced lung injury [2,4,5]. Its pathological hallmarks include impaired alveolarization and abnormal pulmonary vascular development, giving rise to heterogeneous structural and functional phenotypes with highly variable long-term respiratory outcomes [4,5].

Although recent respiratory support-based classifications have improved the prognostic value of BPD diagnosis, they remain indirect clinical definitions that provide limited information on the biological heterogeneity and longitudinal evolution of lung injury [2,3,4,5]. Likewise, currently available prediction models, which mainly rely on clinical variables such as gestational age, birth weight, oxygen exposure, and ventilator settings, are influenced by local clinical practices and generally provide only a binary prediction of BPD at 36 weeks’ postmenstrual age without capturing the dynamic progression of lung injury over time [3,4].

Despite continuous improvements in neonatal care, BPD remains highly prevalent among extremely preterm infants, particularly those born before 28 weeks of gestation, and continues to represent a major determinant of long-term respiratory morbidity into childhood and beyond [6,7,8,9,10]. These limitations have stimulated increasing interest in complementary bedside imaging techniques capable of providing longitudinal information on peripheral lung abnormalities while remaining safe and suitable for repeated use in this vulnerable population [11,12].

Chest radiography remains the conventional first-line imaging modality but has limited sensitivity for subtle peripheral lung abnormalities and exposes infants to cumulative ionizing radiation when repeated over time [11,12]. Computed tomography provides superior structural characterization but is unsuitable for routine serial assessment because of radiation exposure, the need to transport clinically fragile infants, and limited bedside feasibility [11]. By contrast, lung ultrasound (LUS) is a radiation-free, repeatable bedside technique that can be readily integrated into routine neonatal care using standardized acquisition protocols [12,13,14].

Because ultrasound waves are largely reflected by normally aerated lung, LUS primarily interrogates the pleural surface and the immediately adjacent subpleural lung, providing indirect information on peripheral loss of aeration, pleural line abnormalities, small subpleural consolidations, and vertical artifacts associated with interstitial involvement [15,16,17]. These characteristics have established LUS as an increasingly valuable adjunctive imaging modality for early risk stratification, longitudinal monitoring, and therapeutic guidance in infants at risk of BPD.

**Figure 1:**
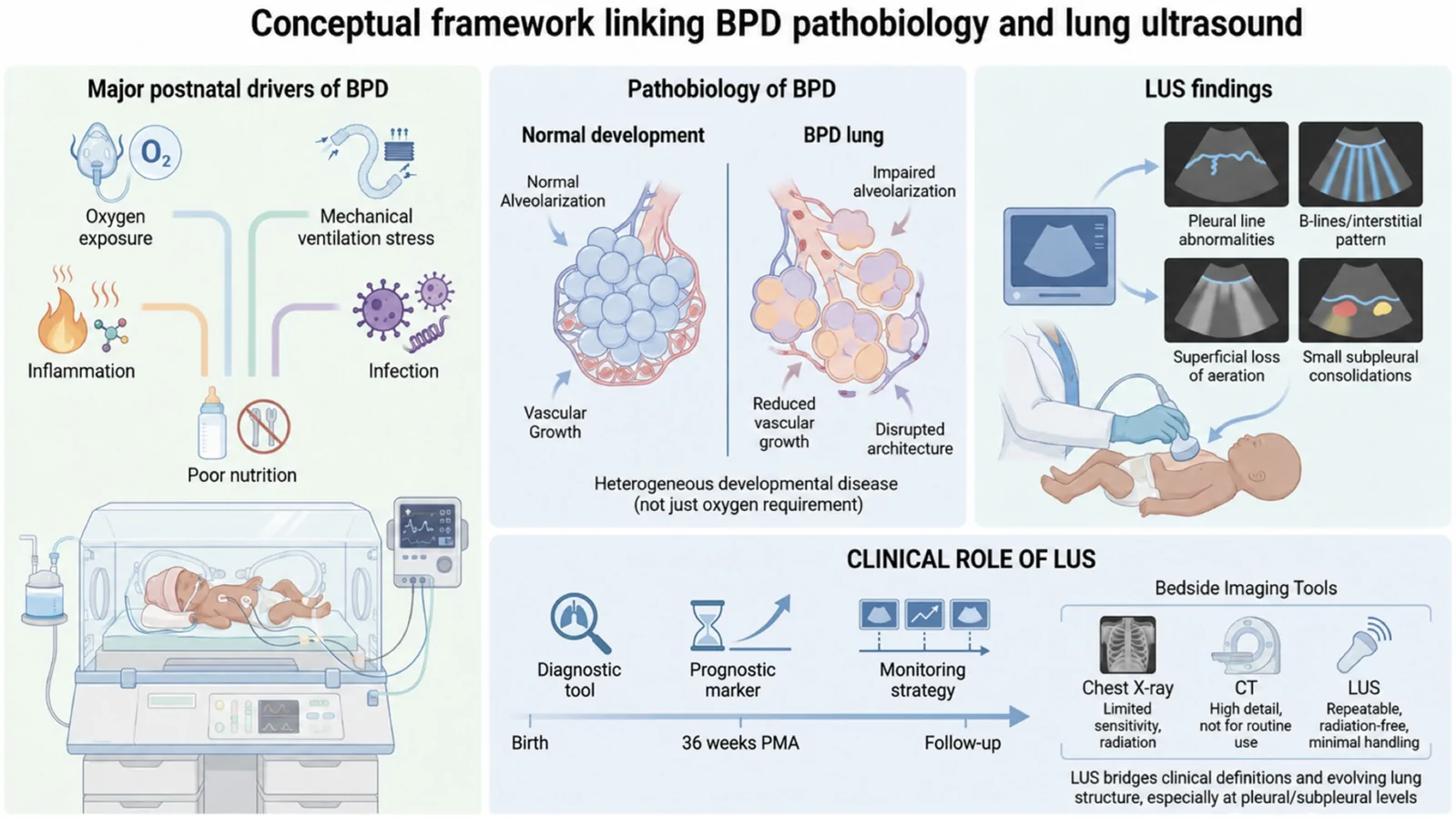
A conceptual framework linking the pathobiology of BPD with the clinical application of LUS. Prematurity-associated postnatal insults, including oxygen exposure, mechanical ventilation, inflammation, infection, and poor nutrition, disrupt normal alveolar and pulmonary vascular development, leading to the heterogeneous phenotype of modern BPD. Because LUS interrogates the pleural surface and adjacent subpleural lung, it identifies indirect sonographic signs of peripheral lung injury, including pleural line abnormalities, vertical artifacts (B-lines), superficial loss of aeration, and small subpleural consolidations. These characteristics support the use of LUS as a bedside imaging modality for bedside assessment, early risk stratification, and longitudinal monitoring throughout the neonatal course. BPD, bronchopulmonary dysplasia; CT, computed tomography; LUS, lung ultrasound; PMA, postmenstrual age. Figure created using FigureLabs https://www.figurelabs.ai/ accessed on 10 July 2026.

This narrative review examines the current evidence regarding the role of LUS in BPD, examining its value as a diagnostic adjunct, an early prognostic marker, and a bedside monitoring strategy. Particular emphasis is placed on the pathophysiological basis of LUS findings, current evidence supporting standardized clinical application, and future developments, including artificial intelligence and personalized imaging approaches. Figure 1 summarizes the pathobiological rationale and proposed clinical applications of LUS in infants with BPD.

## 2. Materials and Methods

This narrative review was designed to provide a structured overview of the current evidence regarding the role of LUS in BPD.

The conduct and reporting of this review were guided by the Scale for the Assessment of Narrative Review Articles (SANRA), which provided a framework to enhance transparency in formulating the review question, conducting the literature search, selecting evidence, applying scientific reasoning, and presenting the available evidence in a balanced manner.

A structured literature search was performed using PubMed. The primary search focused on studies published between January 2010 and June 2026. Earlier landmark publications were also considered when deemed essential to provide historical context on the evolution of BPD definitions, neonatal thoracic imaging, or the development of lung ultrasound in neonatal respiratory care.

The search strategy combined controlled vocabulary (including MeSH terms, where applicable) and free-text keywords related to BPD, prematurity, neonatal respiratory disease, LUS, prediction, monitoring, and respiratory outcomes. Representative Boolean search strings included (“bronchopulmonary dysplasia” AND “lung ultrasound”) and (“preterm infant” AND “lung ultrasound score” AND “bronchopulmonary dysplasia”). Additional searches were performed using combinations of these terms when appropriate. Reference lists of relevant publications were also manually screened to identify additional studies not retrieved through the electronic search.

Eligible publications included human studies involving preterm infants or neonates at risk of or diagnosed with BPD, including prospective and retrospective observational studies, systematic reviews and meta-analyses, clinical guidelines, and technical or methodological papers relevant to neonatal lung ultrasound. Studies were considered eligible if they evaluated LUS in relation to BPD diagnosis, early prediction, longitudinal monitoring, disease severity, respiratory support requirements, oxygen requirement, or pulmonary outcomes. Only articles published in English were considered.

Animal studies, preclinical investigations, single-case reports, and publications not relevant to the role of LUS in BPD or prematurity-related respiratory disease were excluded. Studies focusing exclusively on imaging modalities other than LUS were also excluded unless they provided important comparative or contextual information.

The electronic search was last updated on 30 June 2026. The electronic search retrieved 102 records; these records were evaluated in two stages: initial title and abstract screening, followed by full-text assessment according to the eligibility criteria described above. Potentially eligible articles were discussed among the authors, and disagreements were resolved by consensus. The final reference list includes 69 publications.

Because this is a narrative and not a systematic review, no formal risk-of-bias instrument was applied and no quantitative synthesis was performed. Nevertheless, the relevance and methodological strength of each included study were appraised qualitatively by at least two authors according to prespecified considerations: study design and whether the analysis was prospective; sample size and number of participating centers; whether the scanning protocol and scoring system were prespecified and reported in full; whether the timing of LUS assessment was standardized; the definition of BPD applied and the postmenstrual age at which the outcome was ascertained; and whether measures of predictive performance were reported with confidence intervals. Greater interpretative weight was given to prospective multicenter studies with prespecified protocols and explicitly stated BPD definitions, and to systematic reviews with meta-analysis. Studies with small sample sizes, retrospective designs, or unreported protocol details are identified as such in the accompanying text, and their findings are presented as preliminary. Disagreements in appraisal or interpretation were resolved by discussion until consensus was reached.

The selected evidence was synthesized narratively around three clinically relevant questions: (1) whether LUS serves as a diagnostic adjunct for BPD-related lung abnormalities; (2) whether LUS functions as an early prognostic marker for the development or severity of BPD; and (3) whether serial LUS assessment contributes to longitudinal bedside monitoring during neonatal intensive care and follow-up.

## 3. Pathophysiological Basis of Lung Ultrasound Findings in Bronchopulmonary Dysplasia

**Figure 2:**
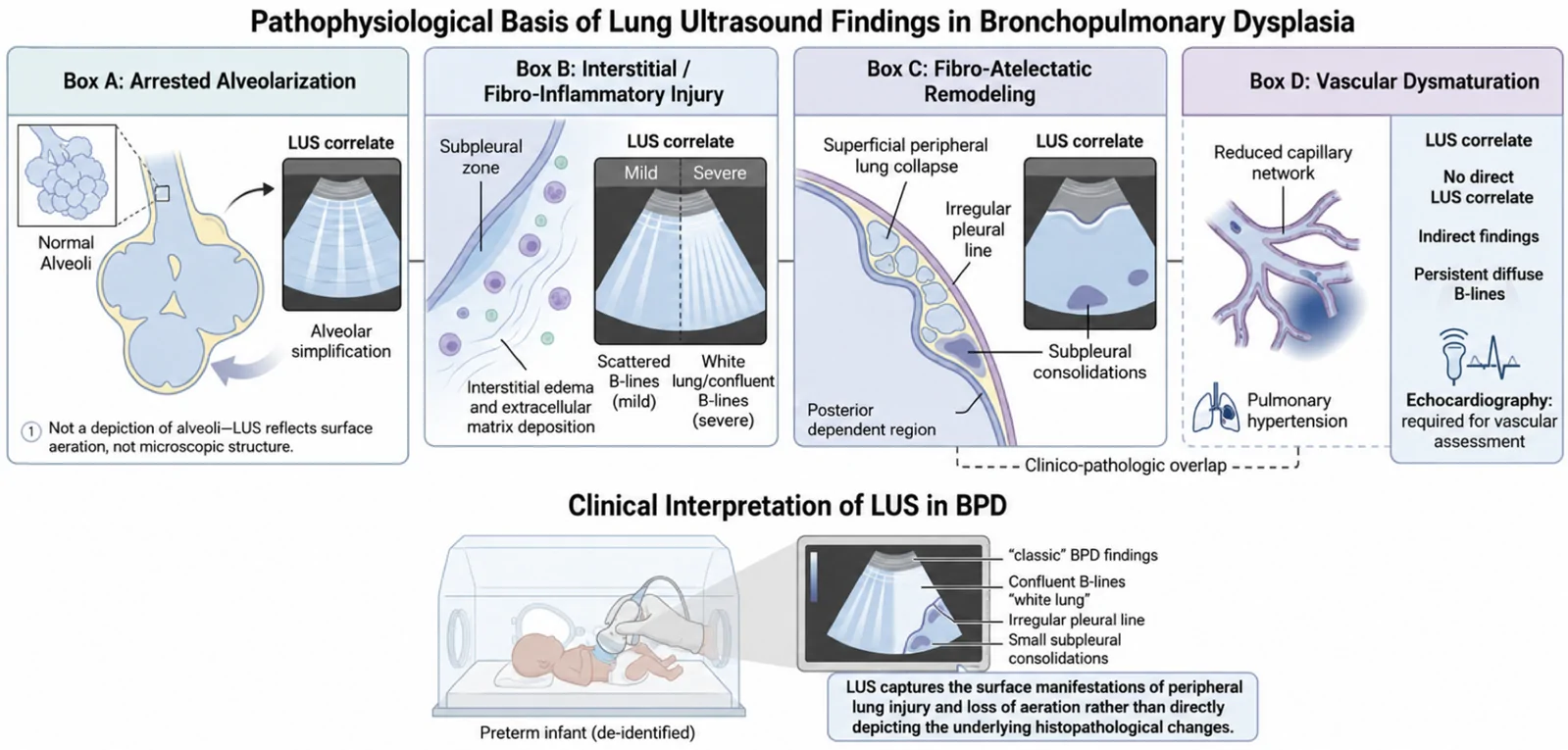
The pathophysiological basis of lung ultrasound findings in bronchopulmonary dysplasia. The principal pathophysiological mechanisms underlying BPD give rise to characteristic but indirect sonographic patterns at the pleural and subpleural lung surface. Arrested alveolarization contributes to peripheral loss of aeration and attenuation of the normal A-line pattern with multiple or confluent B-lines. Interstitial and fibro-inflammatory injury is associated with scattered or confluent B-lines according to the degree of peripheral aeration loss. Fibro-atelectatic remodeling is reflected by pleural line irregularities, pleural thickening, and small subpleural consolidations, predominantly in dependent lung regions. By contrast, vascular dysmaturation has no specific LUS correlate, with ultrasound detecting only indirect consequences of vascular dysfunction, such as diffuse B-lines related to altered pulmonary fluid balance. Overall, LUS should be interpreted as a bedside tool that captures the surface manifestations of peripheral lung injury rather than directly depicting the underlying histopathological changes. BPD, bronchopulmonary dysplasia; LUS, lung ultrasound. Figure created using FigureLabs https://www.figurelabs.ai/ accessed on 10 July 2026.

BPD results from the interaction between interrupted lung development caused by preterm birth and multiple postnatal inflammatory, oxidative, mechanical, and infectious insults acting on the immature lung [4,18,19]. In extremely preterm infants, alveolarization and pulmonary vascular development remain physiologically incomplete, rendering the developing peripheral lung particularly vulnerable to impaired septation, abnormal angiogenesis, and extracellular matrix remodeling [4,20,21]. Although BPD is biologically heterogeneous, four pathophysiological components are particularly relevant for the interpretation of LUS findings: arrested alveolarization, interstitial/fibro-inflammatory injury, fibro-atelectatic remodeling, and vascular dysmaturation [4,20,22]. Each of these mechanisms contributes differently to the sonographic appearance of the lung, providing the pathophysiological basis for interpreting LUS findings in infants with evolving BPD (Figure 2). Because LUS interrogates only the pleural surface and the immediately adjacent subpleural lung, these relationships are necessarily indirect and should be interpreted within the anatomical limitations of the technique.

Arrested alveolarization is the hallmark of modern BPD [4,20,22]. Histologically, it is characterized by fewer and larger alveoli, reduced secondary septation, and a smaller effective surface area for gas exchange [4,21]. Because ultrasound does not directly visualize the alveoli, LUS cannot be considered a surrogate marker of alveolar histology [15,16]. However, when alveolar simplification extends to the subpleural lung and is accompanied by uneven aeration, edema, or inflammation, attenuation of the normal A-line pattern and the appearance of multiple or confluent B-lines may occur [15,17]. In clinical practice, diffuse bilateral B-lines should be interpreted as indicators of peripheral loss of aeration in the appropriate clinical context rather than specific markers of arrested alveolarization, as they may also reflect edema, inflammation, or a combination of these processes [14,17].

Interstitial and fibro-inflammatory injury in BPD is characterized by inflammatory cell infiltration, increased lung water, extracellular matrix deposition, and thickening of the subpleural interstitium [18,20,22]. The subpleural compartment is particularly relevant for LUS because vertical artifacts arise when the ultrasound beam encounters altered air–fluid interfaces immediately beneath the pleural surface [12,15,16]. Consequently, scattered B-lines are generally associated with mild-to-moderate subpleural interstitial involvement, whereas confluent B-lines or a white-lung pattern reflect more extensive loss of peripheral aeration [15,16,17]. In evolving BPD, these sonographic findings are biologically plausible because interstitial inflammation frequently coexists with impaired vascular and lymphatic fluid clearance in the structurally immature lung [4,18,22]. Accordingly, confluent B-lines should be interpreted as sonographic correlates of superficial interstitial–alveolar derangement rather than disease-specific markers of BPD, and always in conjunction with the overall clinical context [4,14,17].

Fibroproliferative and fibro-atelectatic remodeling is less prominent in modern BPD than in the classic pre-surfactant form of the disease but may still occur, particularly in severe cases and following prolonged exposure to mechanical ventilation [4,5,22]. Histologically, fibroproliferative remodeling is characterized by superficial fibrosis, persistent inflammation, and focal atelectatic collapse, resulting in distortion of the pleural surface and adjacent subpleural lung. Consequently, LUS may demonstrate pleural line irregularity, pleural thickening, and small subpleural consolidations [14,15,17]. These consolidations are thought to represent focal peripheral atelectasis, inflammatory collapse, or scar-like remodeling immediately beneath the pleura rather than deep parenchymal lesions [14,16,17]. Their predominance in posterior and dependent lung regions is biologically plausible, reflecting the combined effects of gravity, secretion retention, ventilation heterogeneity, and prolonged respiratory support in infants with evolving chronic lung disease [14,17]. Accordingly, pleural line abnormalities and small subpleural consolidations should be interpreted as sonographic correlates of chronic superficial remodeling rather than disease-specific findings, always in conjunction with the infant’s clinical condition and stage of disease evolution [14,17].

Vascular dysmaturation is a key component of BPD pathobiology, characterized by dysmorphic vascular growth, reduced capillary density, and abnormal pulmonary vascular reactivity, all of which contribute to impaired gas exchange and an increased risk of pulmonary hypertension [4,20,23]. Unlike the other pathophysiological components of BPD, no validated or pathognomonic LUS finding directly reflects pulmonary vascular remodeling [12,14,17]. Instead, LUS is more likely to detect indirect consequences of vascular dysfunction, including interstitial fluid accumulation and secondary peripheral loss of aeration [12,15,16]. Accordingly, persistent diffuse B-lines may suggest a hemodynamic or fluid-related contribution but should not be interpreted as specific markers of pulmonary vascular disease. Comprehensive evaluation of the vascular phenotype still relies on echocardiography and integrated cardiopulmonary assessment [4,11,23]. Therefore, LUS should be regarded as a complementary bedside tool for monitoring the pulmonary consequences of vascular dysfunction rather than a substitute for hemodynamic assessment [4,11,23].

Taken together, these pathophysiological mechanisms provide a pathophysiological framework for interpreting LUS findings in BPD [4,14,17]. Arrested alveolarization, interstitial and fibro-inflammatory injury, fibro-atelectatic remodeling, and vascular dysmaturation each contribute differently to the sonographic appearance of the lung, explaining the heterogeneity of LUS findings observed in affected infants. Importantly, these sonographic patterns should be interpreted as indirect correlates of superficial lung injury rather than direct representations of the underlying histopathological changes. Accordingly, the principal value of LUS in BPD does not lie in identifying specific histopathological lesions but in capturing the dynamic surface manifestations of peripheral lung injury and loss of aeration, thereby providing clinically meaningful information for bedside assessment and longitudinal monitoring [14,17].

## 4. Imaging Modalities in BPD

### 4.1. Chest Radiography

Chest radiography remains the first-line imaging modality in neonatal intensive care because of its widespread availability and ease of use. However, its diagnostic value in BPD is limited by structural superimposition, poor sensitivity for subtle peripheral lung abnormalities, limited correlation with the underlying parenchymal disease, and cumulative exposure to ionizing radiation when serial examinations are required [24].

### 4.2. Computed Tomography

Computed tomography (CT) provides superior structural characterization of the lung and supports validated imaging severity scores. Nevertheless, despite the availability of low-dose protocols (mean effective dose approximately 0.10 ± 0.01 mSv in infants), radiation exposure, the need to transport clinically fragile neonates, and the limited feasibility of repeated examinations substantially restrict its role in routine longitudinal monitoring [24]. Consequently, both techniques have important limitations for serial bedside assessment, reinforcing the rationale for complementary radiation-free imaging modalities such as LUS.

### 4.3. Magnetic Resonance Imaging

Lung magnetic resonance imaging (MRI) is a radiation-free technique that provides detailed structural and functional assessment of the developing lung. Recent ultrashort echo-time free-breathing sequences have shown promising correlations with BPD severity and short-term respiratory outcomes. However, MRI remains expensive, technically demanding, and largely confined to research settings, limiting its applicability for routine bedside assessment in unstable preterm infants [25].

### 4.4. Echocardiography

Echocardiography plays a complementary role by assessing the cardiovascular consequences of BPD, particularly pulmonary hypertension, which cannot be evaluated by LUS. Current AHA/ATS guidelines recommend routine echocardiographic screening in infants with established BPD because of the high prevalence and prognostic relevance of BPD-associated pulmonary hypertension [26]. Early echocardiographic evidence of pulmonary vascular disease is independently associated with subsequent BPD and adverse cardiopulmonary outcomes, highlighting the complementary nature of echocardiography and LUS in the longitudinal assessment of these infants [27,28,29].

Although each imaging modality provides valuable and complementary information, none combines bedside availability, repeatability, and longitudinal assessment of peripheral lung injury as effectively as LUS (Table 1).

### 4.5. Advantages of Lung Ultrasound

Among the currently available imaging modalities, LUS has emerged as the preferred technique for serial bedside assessment of preterm infants. Several scoring systems have been developed to objectively quantify peripheral lung aeration loss and improve the reproducibility of LUS interpretation. The most widely adopted scoring system, originally described by Brat and colleagues [13], grades each of six thoracic regions on a four-point scale ranging from 0 (normal A-line pattern) to 3 (extended consolidation), giving a total score of 0–18. Several variants exist and are not interchangeable: extended protocols add the posterior fields, yielding a ten-zone score, whereas a modified score assesses four regions on a five-point (0–4) scale, giving a total of 0–16 [30]. Throughout this review, unless otherwise stated, “LUS score” refers to the six-zone 0–18 score, and the protocol and score range are specified whenever a different system is discussed. In the original cohort of 130 neonates, the score correlated with oxygenation independently of gestational age and accurately predicted the need for surfactant therapy.

A major strength of LUS is its excellent reproducibility across operators with different levels of experience. In a multicenter post hoc analysis including 629 scans from 155 preterm infants (<34 weeks’ gestation), inter-rater agreement was consistently high for both individual lung regions and the overall LUS score, regardless of operator expertise [31]. Similarly, a modified 0–16 LUS score demonstrated excellent internal consistency and interobserver reliability while showing a strong inverse correlation with oxygenation (SpO_2_/FiO_2_ ratio), supporting its value as a standardized tool for assessing neonatal lung aeration [30].

Beyond its role in the acute management of neonatal respiratory disease, serial LUS assessment has also demonstrated important prognostic value. In a multicenter longitudinal cohort of 147 infants born before 31 weeks’ gestation, gestational age-adjusted LUS scores predicted BPD at 36 weeks’ postmenstrual age from the seventh day of life, with good discrimination (area under the ROC curve 0.826–0.833 on day 7 and 0.834–0.858 on day 14) [14]. Notably, discrimination was achieved only after adjustment for gestational age, indicating that the raw score alone is insufficient for individual risk estimation. These findings support the use of LUS not only as a bedside tool but also as an objective marker for longitudinal risk stratification in infants at high risk of BPD.

The prognostic performance of LUS has subsequently been confirmed by several systematic reviews and meta-analyses. A meta-analysis by Pezza et al. [32] including infants born at ≤32 weeks’ gestation reported pooled sensitivities of 70–80% and specificities of 80–87% for predicting BPD during the first two weeks of life, with a summary ROC AUC of 0.86 at day 14. Similarly, a subsequent meta-analysis including 10 studies (1274 neonates) reported an overall AUC of 0.87, with a sensitivity of 75% and a specificity of 83%, for the prediction of any BPD from LUS performed at day 7 of life; when the outcome was restricted to moderate-to-severe BPD, performance was lower (AUC 0.80, sensitivity 69%, specificity 79%) [33]. These findings are consistent with earlier single-center studies, in which weekly LUS assessment predicted BPD from the first week of life, with optimal thresholds of ≥5 at one week and ≥4 at four weeks of life [34], and with additional meta-analytic evidence demonstrating comparable diagnostic performance for predicting the need for surfactant therapy or respiratory support [35]. Collectively, these data indicate that LUS provides useful early risk stratification and longitudinal monitoring, with a potential but still limited role in guiding selected therapeutic decisions. It should be emphasized that these studies evaluate the predictive accuracy of early LUS for an outcome subsequently defined at 36 weeks’ postmenstrual age, and not the diagnostic accuracy of LUS for established BPD.

Overall, these imaging modalities provide complementary rather than competing information. Unlike the other techniques, LUS uniquely integrates bedside accessibility, radiation-free serial assessment, standardized semiquantitative scoring, and early prognostic information, making it particularly valuable for longitudinal evaluation of infants at risk of BPD. Notably, LUS should be regarded as a complementary imaging modality rather than a replacement for chest radiography, CT, MRI, or echocardiography, as each technique provides distinct structural or functional information.

### 4.6. Limits of Lung Ultrasound

Although LUS exhibits high sensitivity in identifying alterations in pulmonary recruitment and intraparenchymal water content, its diagnostic specificity remains limited when evaluated in isolation [36,37].

The pattern characterized by confluent B-lines, pleural line irregularities, and subpleural consolidations is not pathognomonic for BPD.

Overlapping ultrasound features are frequently encountered in the following:

- Cardiogenic pulmonary edema or overcirculation (e.g., hemodynamically significant PDA): generates a diffuse hydro-aerial pattern with interstitial swelling that mimics acute exacerbations or fluid overload typical of BPD [36,37];
- Neonatal pneumonia and perinatal infections: display consolidations and pleural line disruptions that are difficult to distinguish from the heterogeneous micro-atelectasis seen in advanced BPD [36,37];
- Compressive or obstructive atelectasis may produce focal consolidation or “white lung” appearance without a clear ultrasound signature to differentiate it from the inconsistent aeration characteristic of chronic disease [36,37].

Consequently, LUS must be integrated into a comprehensive, multiparametric framework combining ultrasound findings with clinical history, onset timing, fluid balance, inflammatory markers, and hemodynamic parameters (including point-of-care echocardiography integration).

A further intrinsic limitation of LUS is its operator dependence, which becomes particularly critical in low-volume centers or settings with limited expertise in pediatric/neonatal ultrasonography. The assignment of scores in various LUS scoring systems (e.g., distinguishing between sparse, crowded, or confluent B-lines) suffers from significant inter- and intra-observer variability, particularly during intermediate disease stages [38,39].

Incorrect probe tilting or excessive pressure applied to the preterm infant’s chest can induce artifacts or alter the visualization of the pleural line, leading to over- or underestimation of the severity of lung impairment.

To ensure reproducibility and establish LUS scores as reliable, multicenter tools for clinical guidance, the implementation of standardized training protocols, complemented by certification pathways and ongoing quality control programs, is essential [40]. In this context, the integration of artificial intelligence systems for automated quantitative analysis and B-line counting represents one of the most promising avenues to mitigate operator subjectivity [38,39].

## 5. Current Evidence for Lung Ultrasound in BPD

### Distinguishing Diagnostic, Prognostic, Monitoring, and Treatment-Guiding Roles

Before reviewing the available evidence, it is necessary to distinguish four conceptually and methodologically different roles that LUS may play in BPD, because the literature supports them very unequally and the terms are frequently used interchangeably.

The diagnostic role would require LUS to identify established BPD in an infant at 36 weeks’ postmenstrual age. No study has validated LUS against a reference standard for this purpose, and the sonographic pattern of BPD—confluent B-lines, pleural line irregularity, and small subpleural consolidations—is not disease-specific, being shared with cardiogenic pulmonary edema, infection, and atelectasis. LUS therefore cannot currently be regarded as a diagnostic test for BPD.

The prognostic role, by contrast, accounts for most of the available evidence. Here LUS is performed early in the neonatal course, typically between the third and twenty-eighth day of life, and the outcome is BPD as subsequently defined at 36 weeks’ postmenstrual age by respiratory support criteria. Studies reporting “diagnostic accuracy” in this setting are in fact reporting predictive accuracy for a future outcome, and the two should not be conflated: an infant with a high LUS score on day 7 does not have BPD, but has an increased probability of developing it.

The monitoring role concerns the longitudinal behavior of the score within an individual infant. Serial LUS describes the evolution of peripheral lung aeration over time, and the divergence between infants who do and do not develop BPD is reproducible across cohorts and across BPD definitions. This evidence is descriptive: it establishes that LUS tracks the course of lung injury, not that acting on the trajectory changes it.

The treatment-guiding role is the least supported. Randomized evidence exists only for LUS-guided surfactant administration, and even there the demonstrated benefits concern the timing of treatment and the need for invasive ventilation rather than a reduction in BPD. No randomized trial has evaluated LUS-guided postnatal corticosteroid therapy or LUS-guided extubation.

The question framed in the title of this review is addressed accordingly: on current evidence LUS is best understood as a prognostic marker and a monitoring strategy, rather than as a diagnostic tool for BPD or as an established instrument for guiding treatment.

LUS has evolved from an adjunctive bedside tool into an increasingly validated tool for the prediction and longitudinal assessment of BPD. Its widespread availability, ease of bedside application, absence of ionizing radiation, and excellent reproducibility have facilitated its integration into neonatal clinical practice [41]. Nevertheless, despite the growing body of evidence, standardized international recommendations for the use of LUS in BPD are still lacking. Differences in scanning protocols, timing of examinations, scoring systems, and outcome definitions continue to limit the development of universally accepted clinical guidelines [42].

The first clinical studies investigating LUS in chronic lung disease of prematurity were published only a few years ago and were mainly single-center experiences [42]. Although these studies demonstrated promising diagnostic performance, their relatively small sample sizes limited the generalizability of the findings. More recently, multicenter investigations have substantially strengthened the evidence supporting LUS in BPD.

The first multicenter study, published in 2021, demonstrated that the Lung Ultrasound Score accurately predicts moderate-to-severe BPD from the first days of life, with progressively increasing predictive performance at days 7 and 14 [43]. Importantly, the study also showed that a simplified six-zone anterolateral protocol performed comparably to a more extensive protocol including the posterior regions at most time points, reducing infant handling, although the addition of posterior fields significantly improved diagnostic accuracy on day 14 (*p* = 0.01) [43].

Subsequent multicenter longitudinal studies further expanded these observations by demonstrating that LUS does not merely predict BPD but also reflects the evolving pathophysiology of the disease [14]. In extremely preterm infants, serial LUS assessment closely paralleled respiratory effort, pulmonary gas exchange, and the subsequent development of BPD. Infants who ultimately developed moderate-to-severe BPD exhibited persistently elevated or progressively worsening LUS scores from the second week of life onward, whereas those without BPD showed a progressive decline in LUS values over time [14,44]. These findings support the concept that LUS provides a dynamic bedside assessment of disease evolution rather than a single static prediction of outcome.

The most recent multicenter evidence further consolidated this concept by providing the first detailed longitudinal description of lung pathophysiology during the first weeks of life in infants who subsequently developed moderate-to-severe BPD [44]. In this cohort of 347 extremely preterm infants, serial LUS examinations performed from day 10 through 36 weeks’ postmenstrual age identified a reproducible trajectory characterized by persistent loss of lung aeration, progressive impairment of gas exchange, and increasing respiratory support requirements. Notably, these ultrasound abnormalities evolved independently of the clinical definition used to classify BPD at 36 weeks’ postmenstrual age, including the NIH Consensus, Jensen, and NICHD criteria [3,44,45,46].

Collectively, the currently available evidence identifies three consistent ultrasound features associated with subsequent moderate-to-severe BPD. First, infants who later develop severe disease fail to exhibit the expected postnatal improvement in lung aeration: in the PATH-BPD cohort, in which the earliest assessment was performed on the tenth day of life, lung aeration and oxygenation were already poorer in infants who subsequently developed moderate-to-severe BPD, and the difference widened over time, peaking at 26 days [44]. Second, their longitudinal trajectory diverges from that of infants with mild or no BPD, with stabilization or progressive worsening of LUS scores from the second week onward rather than the expected improvement in lung aeration [14,44]. Third, these ultrasound trajectories remain remarkably consistent across different clinical definitions of BPD, suggesting that LUS captures the underlying biological evolution of lung injury rather than merely reflecting the diagnostic criteria applied at 36 weeks’ postmenstrual age [44].

Although these advances have substantially strengthened the evidence supporting LUS in BPD, several challenges remain. Future research should focus on harmonizing acquisition protocols, scoring systems, examination timing, and clinically relevant outcome measures while validating LUS-guided management strategies in large international multicenter cohorts.

The main characteristics and findings of these multicenter studies are summarized in Table 2.

## 6. A Proposed Pragmatic Framework for the Clinical Use of Lung Ultrasound

The following section outlines a pragmatic framework for the use of LUS in infants at risk of BPD, derived from the protocols, schedules, and populations employed in recent multicenter studies [14,43,44]. It is offered as a proposal to support consistency in clinical practice and in future research, and not as a set of recommendations. Two limitations should be borne in mind throughout. First, formal international guidelines for the use of LUS in BPD do not exist. Second, and more importantly, the supporting evidence derives almost entirely from observational prognostic studies; with the partial exception of LUS-guided surfactant administration, discussed in Section 6.5, no randomized trial has demonstrated that management guided by LUS improves clinically relevant outcomes in this population, and none has shown a reduction in the incidence of BPD. The elements described below should therefore be regarded as reasonable and evidence-informed defaults for standardizing practice and designing future studies, rather than as validated standards of care.

### 6.1. Scanning Protocols in Current Use

The six-zone anterolateral protocol is the approach most frequently adopted in studies of preterm infants at risk of BPD, and is the one for which comparative data are available [13,43,47]. In a multicenter diagnostic accuracy study, adding the posterior fields did not significantly improve prediction of moderate-to-severe BPD at most time points, although it did so on day 14 [43]. Because posterior scanning requires additional handling of clinically fragile infants, the anterolateral protocol represents a pragmatic default for serial assessment, with the posterior fields added when the additional information is likely to be clinically relevant—for example in unexplained clinical deterioration, discordance between clinical and sonographic findings, or suspected dependent atelectasis [47]. This is a practical tradeoff between diagnostic yield and infant handling rather than a validated recommendation, and the choice of protocol should be reported explicitly in future studies to allow for comparison of results. It should also be noted that a meta-analysis comparing the classical six-area and the extended ten-area scores found no significant difference in predictive accuracy at any time point [32], so the incremental value of posterior scanning remains uncertain.

**Figure 3:**
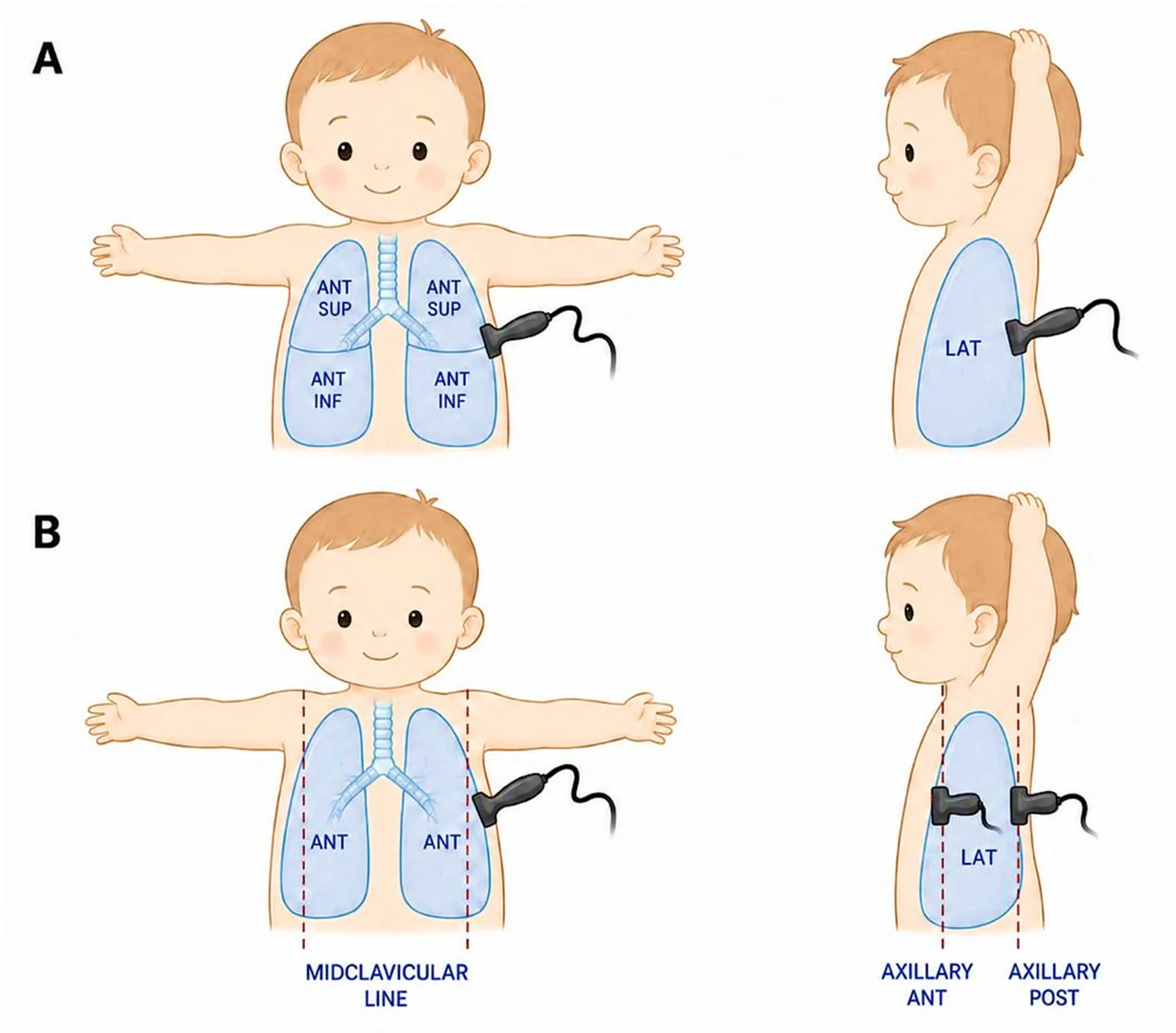
Six-zone lung ultrasound scanning protocols proposed for the assessment of preterm infants at risk of BPD. (**A**) The original six-zone protocol described by Brat et al. [13], in which each anterior hemithorax is divided into an upper (anterior superior) and a lower (anterior inferior) region by the intermammary line, while the lateral region is examined between the anterior and posterior axillary lines. (**B**) The alternative six-zone protocol proposed by Raimondi et al. [48], in which each anterior hemithorax is divided into medial and lateral regions by the midclavicular line, with the lateral region defined between the anterior and posterior axillary lines. Both protocols assess six lung regions (three per hemithorax) and have shown comparable diagnostic performance for lung ultrasound evaluation in preterm infants. The six-zone anterolateral approach is the protocol most frequently used for serial assessment of infants at risk of BPD, and the posterior fields are added in selected clinical situations, as discussed in Section 6.1. Figure created using FigureLabs https://www.figurelabs.ai/ accessed on 12 July 2026.

The two most widely adopted six-zone scanning protocols are illustrated in Figure 3. Both evaluate three regions on each hemithorax (two anterior and one lateral), differing only in the subdivision of the anterior chest. In the original protocol described by Brat et al. [13], the anterior hemithorax is divided into upper and lower regions by the intermammary line, whereas Raimondi et al. [48] proposed medial and lateral anterior regions separated by the midclavicular line. In both approaches, the lateral region is bounded by the anterior and posterior axillary lines. Importantly, these minor anatomical differences have not been shown to influence the overall clinical performance of LUS, allowing either protocol to be used within a standardized scanning strategy.

### 6.2. Lung Ultrasound Scoring and Image Interpretation

**Figure 4:**
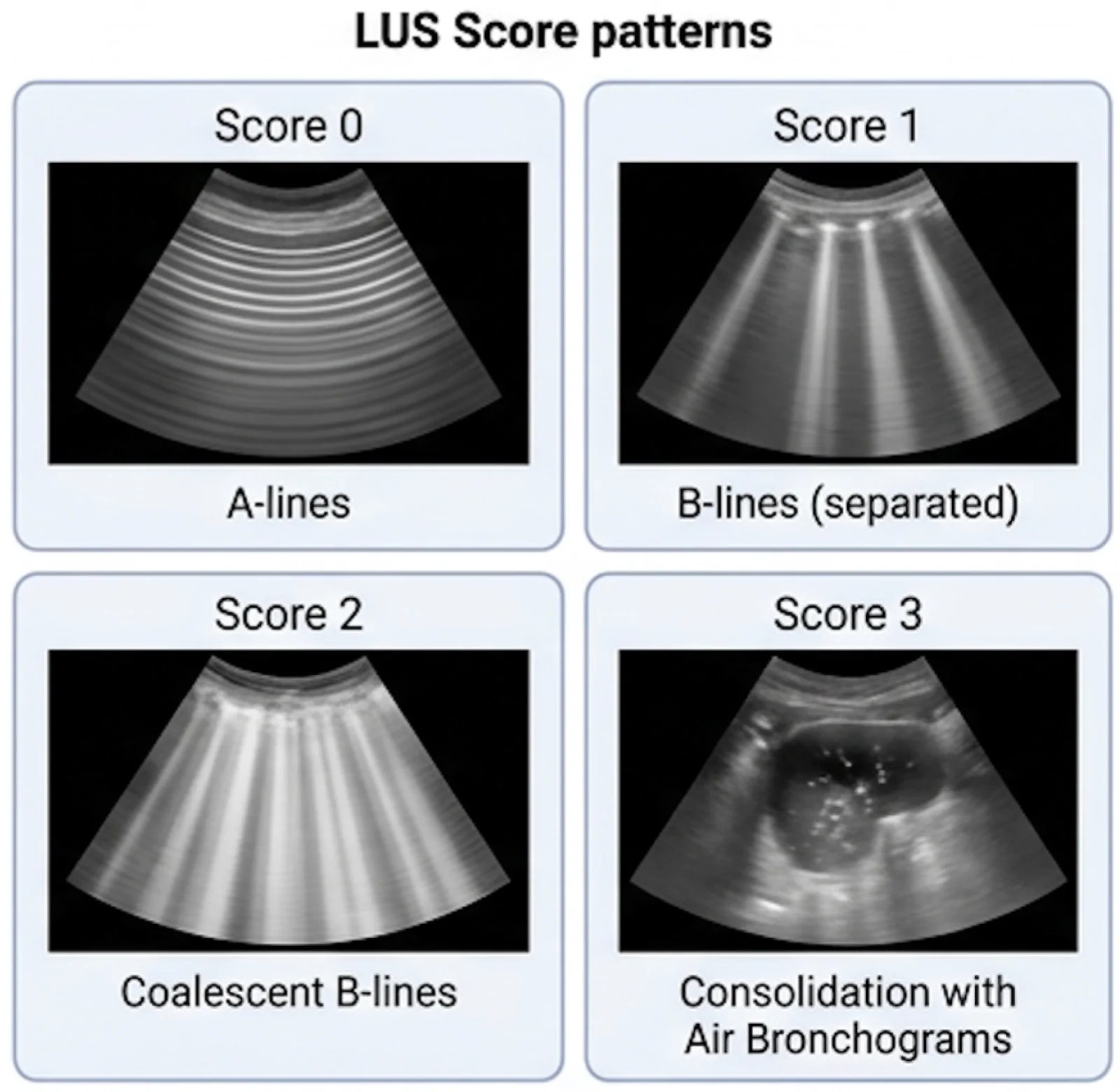
Representative lung ultrasound patterns corresponding to the Lung Ultrasound Score. Representative sonographic appearance of the four semi-quantitative LUS score categories (scores 0–3) to assess peripheral lung aeration in preterm infants. Figure created using FigureLabs https://www.figurelabs.ai/ accessed on 7 July 2026.

Semi-quantitative assessment of lung aeration is based on the Lung Ultrasound Score, which assigns a score from 0 to 3 to each of the six examined lung regions according to the degree of aeration loss. The total score therefore ranges from 0, indicating a normally aerated lung in all examined regions, to 18, corresponding to diffuse loss of aeration with extensive pulmonary consolidations [13,49]. Representative ultrasound patterns corresponding to each score are illustrated in Figure 4.

Score 0 corresponds to a normal A-line pattern. Score 1 is characterized by the presence of three or more well-defined B-lines within a single scanning field. Score 2 reflects severe interstitial involvement with crowded or confluent B-lines, with or without small subpleural consolidations. Finally, score 3 indicates extensive pulmonary consolidation with complete loss of regional aeration [13,49].

Importantly, the LUS score should be regarded as a standardized measure of peripheral lung aeration rather than as an isolated diagnostic test, and its interpretation should always be integrated with the infant’s clinical condition.

Beyond its descriptive role, the LUS score carries prognostic information, and several operating points have been proposed. These are summarized, together with the population, scanning protocol, timing, and BPD definition to which each applies, in Table 3.

In the largest multicenter diagnostic accuracy study to date, comprising 832 examinations in 298 infants, a score of ≥8 on the seventh day of life using the anterolateral six-zone protocol predicted moderate-to-severe BPD with a sensitivity of 70%, a specificity of 79%, a positive likelihood ratio of 3.3, and a negative likelihood ratio of 0.38; the authors characterized the resulting accuracy as moderate [43]. In a single-center study of very-low-birth-weight infants scanned weekly, a score of ≥5 at one week of life predicted any BPD (sensitivity 71%, specificity 80%, AUC 0.80), and a score of ≥4 at four weeks of life predicted moderate-to-severe BPD (sensitivity 100%, specificity 80%, AUC 0.89) [34].

The influence of the outcome definition is illustrated particularly clearly by a cohort in which two definitions were applied in parallel to the same day-7 examinations: discrimination for BPD fell from an AUC of 0.87 (95% CI 0.79–0.94) under the NICHD 2001 [2] definition to 0.80 (95% CI 0.70–0.90) under the Jensen 2019 definition [3], in the same infants scanned on the same day [17]. The apparent performance of a LUS score is therefore in part a property of the outcome definition applied to it, and not of the measurement alone.

These figures illustrate why the reported thresholds are not interchangeable. They were derived in populations of different gestational age, using different numbers and locations of scanned regions, at different postnatal ages, and against different definitions of BPD. Pooled estimates are of the same order as those of individual studies, and higher at later time points—meta-analyses report sensitivities of 70–80% and specificities of 80–87% during the first two weeks of life [32], rising to 85% and 86% by day 21 [50]—but they diverge downward when the outcome is restricted to moderate-to-severe BPD, for which pooled sensitivity falls to 69% and specificity to 79% [33]. The discrepancy between individual studies and pooled estimates therefore reflects the outcome definition and the timing of assessment rather than a systematic optimism of the primary reports (Table 4).

Predictive performance is also dependent on gestational age. In infants born at 23–27 weeks, the LUS score added little discriminative information beyond gestational age alone, whereas in infants born at ≥28 weeks a multivariable clinical model incorporating gestational age, sex, hemodynamically significant patent ductus arteriosus, mode of respiratory support, and transcutaneous oxygen tension outperformed the LUS score alone (AUC 0.91 versus 0.77) [51].

The cutoffs reported above should therefore be regarded as study- and protocol-specific operating points rather than universally validated thresholds. They should not be transferred to a different population, scanning protocol, or BPD definition without local verification, and in clinical practice the LUS score is best interpreted as a graded measure of peripheral lung aeration within the overall clinical assessment rather than as a dichotomous test.

### 6.3. Populations in Which Serial Assessment Has Been Evaluated

Published studies of serial LUS monitoring have primarily focused on preterm infants at increased risk of developing BPD, in whom longitudinal assessment of lung aeration has been evaluated for early risk stratification and disease monitoring [14,43,44]. Evidence supporting routine serial LUS assessment across the broader neonatal intensive care population remains limited. Based on the available multicenter evidence, the populations in which serial LUS assessment has been evaluated, and in which it is therefore most likely to be informative, are as follows:

- Extremely preterm infants, especially those born at ≤30 weeks’ gestation and, in particular, ≤28 weeks’ gestation [14,43,44].
- Extremely low-birth-weight infants, generally with a birth weight <1000 g, or very low-birth-weight infants at high respiratory risk, depending on local clinical protocols [14,43,44].
- Infants requiring prolonged respiratory support, including invasive mechanical ventilation, continuous positive airway pressure (CPAP), or high-flow nasal cannula therapy beyond the first week of life, as these patients have the highest probability of developing evolving BPD [14,43,44].

In lower-risk preterm infants or in those with rapidly improving respiratory status, LUS should be performed according to the clinical indication rather than as part of a predefined monitoring protocol. These are the inclusion criteria of the cohorts in which the prognostic value of LUS has been demonstrated, and not validated indications for monitoring. Whether serial LUS assessment should be performed in a given infant remains a clinical judgment, and no study has compared a strategy of protocolized serial LUS with clinically indicated assessment.

### 6.4. Timing of Serial Assessment in Published Studies

The assessment schedules used in longitudinal multicenter studies are summarized below. They reflect the designs of those studies rather than a validated protocol, and the timing of examinations should always be adapted to the infant’s clinical condition [14,43,44].

Early predictive assessments have been evaluated between days 3 and 7 of life. Among these time points, day 7 has shown consistent predictive performance across studies; in one multicenter cohort, an anterolateral score of ≥8 on day 7 identified infants at increased risk of moderate-to-severe BPD, with a sensitivity of 70% and a specificity of 79% [43].

Assessments performed between days 10 and 14 have also been evaluated for longitudinal risk stratification. During this period, infants who subsequently develop moderate-to-severe BPD begin to show a clear divergence in LUS trajectories compared with those demonstrating physiological pulmonary recovery [14,44].

Later LUS assessments, particularly between days 21 and 28, have been evaluated as markers of evolving chronic lung disease, with higher scores during this period associated with persistent lung aeration abnormalities and subsequent BPD severity [44]. The progressive improvement in the predictive performance of LUS reflects the biological evolution of the immature lung. During the first postnatal days, residual fetal lung fluid, surfactant deficiency, and respiratory distress syndrome produce ultrasound patterns that may overlap with those of evolving BPD, reducing diagnostic specificity. As lung fluid clearance progresses and transient respiratory disease resolves, LUS findings become increasingly representative of chronic peripheral lung injury. Accordingly, pooled predictive accuracy improves from a sensitivity of 75% and a specificity of 74% on days 1–3 to a sensitivity of 85% and a specificity of 86% by day 21 [50].

### 6.5. Lung Ultrasound and Therapeutic Decisions: What the Evidence Does and Does Not Support

LUS has been proposed as an aid to several therapeutic decisions in neonatal intensive care, including surfactant administration, postnatal corticosteroid therapy, and extubation. The strength of the underlying evidence differs substantially between these applications, and they are therefore considered separately.

Surfactant administration. This is the only application for which randomized evidence exists. During the first hours of life, elevated LUS scores reflect surfactant deficiency and diffuse alveolar collapse, and predefined thresholds identify infants likely to require surfactant replacement; the reported thresholds again depend on the protocol and population, ranging from ≥4 using the anterolateral six-zone score in infants below 34 weeks’ gestation [13] to >8 using a six-region score that includes the posterior fields [52]. Three randomized controlled trials have evaluated LUS-guided surfactant administration. In the ULTRASURF trial (*n* = 56, infants ≤ 32 weeks’ gestation), LUS-guided treatment led to substantially earlier administration (median 1 versus 6 h, *p* < 0.001) and lower subsequent oxygen requirements [53]. In a more recent trial (*n* = 89, infants < 32 weeks’ gestation), administration of surfactant when a six-region score including the posterior fields exceeded 8 led to earlier treatment (median 60 versus 87.5 min, *p* = 0.017), a lower rate of invasive ventilation (19.6% versus 46.5%, *p* = 0.007), and a shorter duration of ventilation [54]. In a randomized controlled pilot study by Wu and Su (*n* = 82 preterm infants with RDS), LUS score-directed surfactant treatment did not significantly reduce the duration of mechanical ventilation, supplemental oxygen requirement, or NICU stay compared with conventional clinical–radiological assessment; notably, BPD incidence was identical in the two groups (4.9% in each group) [55]. Importantly, none of these trials demonstrated a reduction in BPD, and the available randomized evidence remains insufficient to establish an effect of LUS-guided surfactant administration on BPD incidence. LUS may therefore facilitate earlier and more targeted surfactant administration through minimally invasive techniques such as Less Invasive Surfactant Administration or Minimally Invasive Surfactant Therapy. Although some randomized studies have reported earlier treatment and reduced short-term respiratory support requirements, these benefits have not been consistently demonstrated across trials, and no reduction in BPD has been established.

Postnatal corticosteroid therapy. No interventional evidence supports the use of LUS to guide postnatal corticosteroid therapy. Observational studies show that infants who develop moderate-to-severe BPD have persistently elevated LUS scores during the second and third weeks of life, and it has been suggested that this information might contribute to the identification of candidates for corticosteroid treatment or to the assessment of treatment response. However, no study has evaluated whether initiating, withholding, or modifying corticosteroid therapy on the basis of LUS findings alters any clinical outcome, and no threshold has been derived or validated for this purpose. The decision to initiate postnatal corticosteroids should continue to be based on established clinical criteria and on the balance of respiratory benefit against neurodevelopmental risk. Any use of serial LUS in this context should at present be regarded as descriptive monitoring of lung aeration rather than as a determinant of treatment.

Extubation. Extubation failure in extremely preterm infants is associated with adverse outcomes: in a systematic review of 120 studies, extubation failure was associated with death and/or BPD with a pooled odds ratio of 4.7 (95% CI 2.84–7.76) [56], and in a secondary analysis of a large multicenter cohort, reintubation within five days carried an adjusted odds ratio of 3.06 for BPD [57]. Improving the prediction of extubation readiness is therefore clinically relevant. Evidence for LUS in this role, however, remains limited to observational data. In a prospective study of 77 preterm infants, the mean pre-extubation LUS score was higher in those who failed extubation (7.0 ± 2.75) than in those extubated successfully (4.89 ± 1.81), and a threshold of >6 predicted extubation failure with a sensitivity of 78.5% and a specificity of 67.7% [58]—accuracy insufficient to determine clinical decisions in isolation. No randomized trial has evaluated LUS-guided extubation. LUS may thus contribute additional information to the assessment of extubation readiness alongside clinical and ventilatory parameters, but it should not be used as a decision rule, and the thresholds reported to date require prospective validation.

Taken together, these applications illustrate both the potential of LUS as a bedside tool for individualizing respiratory management and the current limits of the supporting evidence. With the partial exception of surfactant administration, the available data establish associations between LUS findings and clinical outcomes rather than demonstrating that LUS-guided management improves those outcomes. Interventional studies designed with patient-relevant primary endpoints are required before LUS can be incorporated into therapeutic algorithms.

## 7. Artificial Intelligence and Future Perspectives

Artificial intelligence (AI) is emerging as one of the most promising developments in neonatal LUS, with the potential to improve the accuracy, reproducibility, and clinical utility of LUS in infants at risk of BPD. Rather than replacing the clinician, AI is intended to complement clinical expertise by reducing operator dependency, standardizing image interpretation, and facilitating the integration of ultrasound findings with other clinical data [59,60].

### 7.1. Current Artificial Intelligence Models for Neonatal Lung Ultrasound

Several neonatal applications have been reported. In a multicenter prospective diagnostic study of 62 newborns (mean gestational age 36 ± 2 weeks) with early respiratory distress, Perri et al. [60] developed an artificial intelligence model to classify neonatal LUS patterns and predict the need for continuous positive airway pressure or surfactant therapy. The model achieved area under the receiver operating characteristic curves (AUROCs) of 0.88 and 0.80 for continuous positive airway pressure at the first two assessment times, and 0.84 and 0.89 for surfactant therapy, respectively.

Fatima et al. [59] evaluated two deep learning approaches using 70 LUS examinations from 34 neonates, with annotations provided by three expert operators. The transfer-learning frame-to-video strategy achieved 77% accuracy and demonstrated moderate agreement with the experts, whereas direct video classification achieved 72% accuracy and substantial agreement.

These published systems should be considered research prototypes. They focused on neonatal respiratory-pattern interpretation and early respiratory-support decisions rather than BPD diagnosis or prediction. Furthermore, neither study provided prospective external validation in an extremely preterm population at risk of BPD [59,60].

Automated calculation of LUS scores represents a promising application of AI. Deep learning architectures can potentially identify the pleural line, detect and quantify B-lines and consolidations, and support automated or semi-automated assignment of regional LUS scores.

Automated scoring has the potential to reduce inter-observer variability and improve the reproducibility of LUS assessment across operators with different levels of experience [59,60]. Comparable architectures have been applied to adult lung ultrasound, where deep learning models localize and grade pulmonary artifacts at frame and video level [61], providing the methodological precedent for neonatal applications.

AI may also improve image acquisition. In adult lung ultrasound, a system providing real-time workflow prompts, landmark identification, and automated capture once diagnostic image quality was reached enabled minimally trained operators—including nurses and technicians without prior ultrasound experience—to obtain diagnostic-quality eight-zone examinations in 98.3% of cases, comparable to those obtained by expert operators [62]. Analogous deep learning systems providing prescriptive probe-positioning guidance have been validated in echocardiography, where operators without prior experience obtained diagnostic-quality images in 98.8% of studies for left ventricular size and function and in 92.5% for right ventricular size [63]. Such acquisition-side technologies address precisely the operator dependence that limits the reproducibility of neonatal LUS scoring.

It must be emphasized, however, that no AI system for real-time acquisition guidance or automated image quality control has been developed or validated in neonatal lung ultrasound. All published neonatal LUS applications of AI operate on images that have already been acquired [59,60]. The extension of acquisition-side AI to extremely preterm infants—in whom the limited thoracic surface and patient instability make examination technically demanding—is therefore a plausible but entirely untested development rather than an established capability.

A further area of development is the integration of ultrasound findings with physiological and clinical variables. Conventional multivariable models have been used to compare and to combine the LUS score with clinical predictors—gestational age, sex, hemodynamically significant patent ductus arteriosus, mode of respiratory support, and transcutaneous oxygen tension—for the prediction of moderate-to-severe BPD [51], and gestational age-adjusted serial LUS scores predict BPD from the first week of life [14]. Machine learning models fusing the LUS score with oxygenation and respiratory indices have been reported in neonatal respiratory distress syndrome, where a random forest classifier achieved an AUC of 0.894 for disease severity, although the outcome was not BPD [64]. Separately, machine learning models using dynamically updated ventilator settings and blood gas values—but no imaging—predict BPD with external validation (AUROC 0.814 at external validation, 0.841 in development and 0.912 in internal validation) [65]. Deep learning models have likewise been developed to predict BPD from chest radiographs [66], although this addresses a different imaging modality and does not inform the use of ultrasound.

No multimodal machine learning model integrating lung ultrasound with clinical and physiological variables for continuously updated BPD risk estimation has yet been published, and none has undergone prospective external validation for this purpose. The convergence of these separate lines of work is a plausible and frequently proposed direction, but it remains a research objective rather than a clinically deployable application [67].

Although these technologies remain largely investigational, early results are encouraging and suggest that AI may further expand the role of LUS from a semiquantitative bedside tool to an objective, standardized, and data-driven decision support tool.

### 7.2. Limitations and Regulatory Considerations

Several significant limitations continue to impede the clinical translation of AI in this field. Most existing studies rely on single-center cohorts with small sample sizes, underscoring the need to validate these models across diverse neonatal populations and ultrasound platforms. Additionally, most commercial AI-integrated ultrasound algorithms have been trained and calibrated primarily on adult or older pediatric populations [68].

Model performance may also decline across different ultrasound devices, probes, acquisition protocols, and institutions. Consequently, BPD-focused development requires multicenter, prospective external validation in extremely preterm infants, comprehensive calibration and subgroup analyses, transparent failure reporting, explainability aligned with the intended clinical task, and assessment of whether clinician–AI collaboration improves patient-relevant outcomes rather than solely image classification accuracy [59,60,69].

For clinical deployment, an AI-assisted LUS system designed to influence diagnosis or treatment may be classified as medical device software. The regulatory pathway depends on the system’s intended purpose and risk classification. Key requirements include risk management, data governance, technical documentation, human oversight, accuracy, robustness, cybersecurity, controlled model updates, and post-market surveillance. Within the European Union, medical-device AI systems requiring third-party conformity assessment are designated as high-risk under Regulation (EU) 2024/1689 [70].

Good Machine Learning Practice principles further mandate the use of representative datasets, separation of training and test sets, clinically relevant testing, evaluation of human–AI team performance, clear user information, and ongoing lifecycle monitoring. Until BPD-specific external validation and relevant regulatory requirements are fulfilled, AI outputs should function as clinician-supervised decision support tools rather than autonomous recommendations [69].

**Figure 5:**
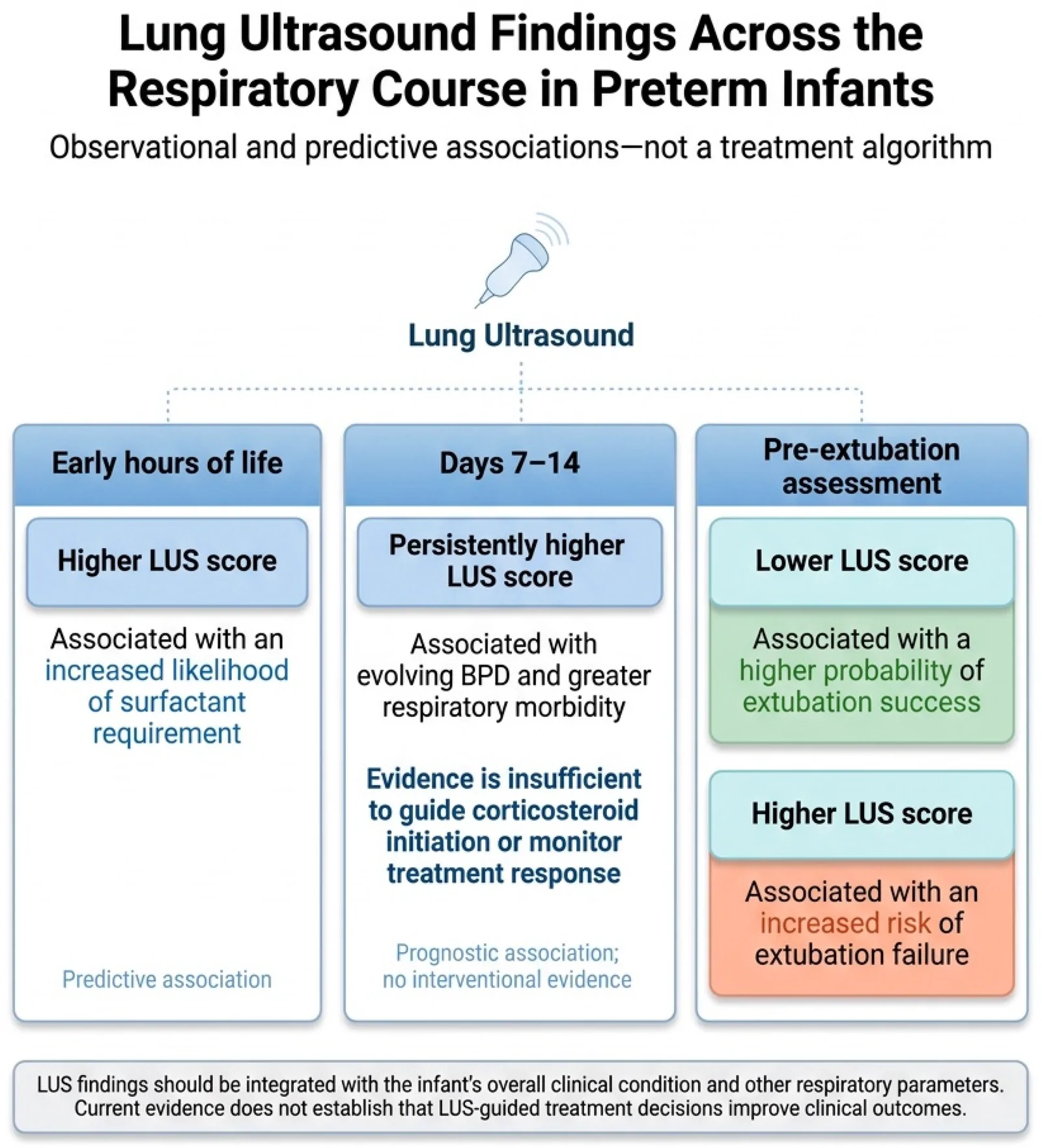
This figure is not a clinical management algorithm. Conceptual overview, informed by current evidence, illustrating associations between serial lung ultrasound (LUS) findings and clinically relevant respiratory outcomes in preterm infants. Elevated LUS scores in the early postnatal period are associated with increased surfactant requirement, while persistently high scores are linked to the development of bronchopulmonary dysplasia. Pre-extubation LUS scores correlate with extubation outcomes. These associations are primarily based on observational, diagnostic, or prognostic studies and should not be regarded as treatment thresholds or clinical management algorithms. Lung ultrasound is intended to complement, not replace, comprehensive clinical assessment. Current evidence does not support the use of LUS to guide corticosteroid initiation or monitoring. Figure created using FigureLabs https://www.figurelabs.ai/ accessed on 10 July 2026.

The current evidence linking LUS findings with clinically relevant respiratory outcomes and potential clinical applications is summarized in Figure 5.

## 8. Conclusions

Lung ultrasound has emerged as a valuable imaging tool that extends beyond the diagnosis of acute neonatal respiratory disease and is increasingly recognized as an important tool for the prediction, monitoring, and longitudinal assessment of BPD. Its unique combination of bedside availability, the absence of ionizing radiation, repeatability, and standardized semiquantitative scoring makes LUS particularly well suited for serial evaluation of preterm infants throughout their clinical course. Its principal established value lies in prognosis and in longitudinal monitoring; LUS has not been validated as a diagnostic test for established BPD, and the thresholds proposed for risk stratification remain specific to the populations and protocols in which they were derived.

The growing body of evidence from multicenter studies supports the use of LUS for early risk stratification and for monitoring the evolution of peripheral lung aeration. Its role in therapeutic decision-making remains, with the partial exception of surfactant administration, unproven: available data establish associations between LUS findings and outcomes rather than demonstrating that LUS-guided management improves them. Nevertheless, LUS should be regarded as an adjunctive imaging technique rather than a replacement for conventional imaging or echocardiography, as each technique provides distinct structural and functional information.

Despite these advances, several challenges remain before LUS can be fully integrated into standardized clinical practice. Future research should focus on harmonizing scanning protocols, scoring systems, examination timing, and clinically relevant outcome measures, while validating LUS-guided management strategies in large international multicenter studies. The integration of artificial intelligence and multimodal predictive models may further enhance the reproducibility, objectivity, and clinical utility of LUS, paving the way toward personalized respiratory care in preterm infants. If future multicenter studies confirm standardized acquisition protocols and demonstrate improved patient outcomes, LUS has the potential to become an integral component of precision respiratory care for preterm infants at risk of BPD.

## Figures and Tables

**Table 1:** Comparison of imaging modalities for the assessment of bronchopulmonary dysplasia. Each modality provides complementary information on different aspects of the disease. Chest radiography remains the first-line examination, CT and MRI offer detailed structural assessment, echocardiography evaluates pulmonary vascular involvement, whereas lung ultrasound (LUS) uniquely enables radiation-free, bedside, serial assessment of peripheral lung abnormalities and longitudinal monitoring.

Imaging Modality	Strengths	Main Limitations	Primary Clinical Application
Chest radiography	Widely available, quick, low cost	Ionizing radiation, poor sensitivity for subtle peripheral abnormalities	Initial assessment
Computed tomography (CT)	Excellent structural detail, validated severity scores	Ionizing radiation, transport of fragile infants, limited serial use	Selected cases/structural characterization
Magnetic resonance imaging (MRI)	Radiation-free, structural and functional assessment	Limited availability, expensive, technically demanding	Advanced structural and functional assessment (research)
Echocardiography	Assessment of pulmonary hypertension and vascular consequences	Limited assessment of lung parenchyma	Vascular/cardiopulmonary assessment
Lung ultrasound (LUS)	Bedside, radiation-free, repeatable, standardized scoring	Interrogates peripheral lung only; operator dependence	Prediction and longitudinal monitoring

**Table 2:** A summary of the key features of the three main multicenter studies mentioned above, detailing the number of enrolled patients, the timing of lung ultrasound execution, the performed scans, and the main results obtained.

Major Multicenter Studies	Enrolled Patients	Timing of LUS Execution	Scanning Technique	Results	BPD Definition
Alonso-Ojembarrena et al. [43]	298 infants	At birth at 3rd, 7th, 14th and 21st DOL	Anterolateral and posterior lung fields	LUS score predicts msBPD from the 3rd DOL with a moderate predictive accuracy. Posterior lung fields improved diagnostic accuracy only at the 14th DOL	Moderate-to-severe BPD
Loi et al. [14]	147 infants	At birth at 7th, 14th and 28th DOL	Anterolateral and posterior lung fields	Gestational age–adjusted LUS scores significantly predict BPD at 7 and 14 DOL. BPD severity and gestational age-adjusted LUS scores are significantly correlated at 7 and 14 DOL Additionally, LUS scores significantly correlate with O2 metrics and work of breathing at any time point	NICHD 2001 [2], at 36 weeks PMA
De Luca et al. [44]	347 infants	At 10th, 21st and 28th DOL 34 and 36 weeks post-menstrual age	Anterolateral lung fields	Lung aeration and oxygenation were always poorer, since 10th DOL in patients with msBPD than in those without it. The difference in lung aeration between patients with and without msBPD increased over time, with a peak at 26th DOL	NIH/NICHD 2001 [2], NICHD 2018 [46] and Jensen 2019 [3], at 36 weeks PMA

**Table 3:** Principal studies evaluating lung ultrasound for the prediction of bronchopulmonary dysplasia. For each study the population, scanning protocol, timing of assessment, and definition of BPD are specified, because the reported thresholds are not interchangeable across these dimensions. a Included as the derivation study of the six-zone 0–18 score used throughout this review; BPD was not an outcome of this study. b Gestational age is not reported in the primary publication; the value shown is as extracted in the meta-analysis by Han et al. [50]. c The primary publication reports areas under the curve for each BPD definition but does not report optimal cutoff values. d The definition of BPD applied is not stated in the primary publication. AUC, area under the receiver operating characteristic curve; BPD, bronchopulmonary dysplasia; BW, birth weight; CPAP, continuous positive airway pressure; DOL, day of life; GA, gestational age; LUS, lung ultrasound; msBPD, moderate-to-severe BPD; NR, not reported; PMA, postmenstrual age; VLBW, very low birth weight.

Study	Design	Sample Size	Gestational Age	Scanning Protocol	Timing of LUS Assessment	BPD Definition	LUS Cutoff	AUC/Sensitivity/Specificity	Principal Findings
Brat et al., 2015 [13] a	Prospective diagnostic accuracy study, single center	130 neonates	All GA; analyzed as <34 and ≥34 weeks	Six anterolateral zones (three per hemithorax, divided by the intermammary and anterior axillary lines); 0–3 per zone; total 0–18	First hours of life, on CPAP, before surfactant	BPD not an outcome	≥4 (GA < 34 weeks)	AUC 0.93 (0.86–0.99); sensitivity 100%, specificity 61%	Derivation of the six-zone 0–18 score; the score correlated with oxygenation independently of GA and predicted the need for surfactant
Abdelmawla et al., 2019 [42]	Cohort study, single center	27 infants (64 examinations)	<30 weeks; median 26 (IQR 25–29); BW 780 g (530–1045)	Three zones per lung (six zones); 0–3 per zone; total 0–18; zone boundaries NR	Between the 1st and 9th postnatal week; median 5 weeks (no fixed time point)	Respiratory support or supplemental oxygen at 36 weeks PMA; any severity	≥6	Sensitivity 76%, specificity 97%; AUC NR. With GA < 27 weeks added: sensitivity 86%, specificity 98%	Median score 9 (6–12) with chronic lung disease versus 3 (1–4) without; small sample and non-standardized timing limit interpretation
Alonso-Ojembarrena & Lubián-López, 2019 [34]	Prospective cohort, single center	59 VLBW infants (21 with BPD)	VLBW infants; GA NR b	Six anterolateral zones; 0–3 per zone; total 0–18; posterior fields not scanned	24 h, 72 h, then weekly until 36 weeks PMA	NIH/NICHD 2001 [2]; reported for both any BPD and msBPD	≥5 at 1 week (any BPD); ≥4 at 4 weeks (msBPD)	1 week: sensitivity 71%, specificity 80%, AUC 0.80. 2 weeks: sensitivity 74%, specificity 100%, AUC 0.93. 4 weeks (≥4, msBPD): sensitivity 100%, specificity 80%, AUC 0.89	The score declined after the first week in infants without BPD but remained elevated until 36 weeks PMA in those with BPD
Aldecoa-Bilbao et al., 2021 [17]	Prospective observational study, single center	89 very preterm infants	≤30 + 6 weeks	Six zones including the posterior fields; per-zone and total range NR	Admission, DOL 7, DOL 28	Two definitions applied in parallel: NICHD 2001 [2] and Jensen 2019 [3], both at 36 weeks PMA	NR c	Day 7, NICHD 2001 [2]: AUC 0.87 (0.79–0.94). Day 7, Jensen 2019 [3]: AUC 0.80 (0.70–0.90). Sensitivity and specificity NR	Discrimination in the same infants on the same day differed according to the BPD definition applied; a combined model (mechanical ventilation > 5 days, oxygen ≥ 7 days, day-7 LUS) reached AUC 0.90 (0.84–0.97)
Loi et al., 2021 [14]	Prospective multicenter longitudinal cohort	147 infants	<31 weeks	Six anterolateral zones; posterior score and extended ten-zone score also computed	DOL 1, 7, 14, 28, and 36 weeks PMA	NICHD 2001 [2] at 36 weeks PMA; msBPD	No single cutoff; gestational age-adjusted score	AUC 0.826–0.833 on day 7 and 0.834–0.858 on day 14 (*p* < 0.0001); sensitivity 71%, specificity 74% on day 7	Prediction required adjustment for gestational age; the score also correlated with oxygenation metrics and work of breathing at all time points
Alonso-Ojembarrena et al., 2021 [43]	Prospective multicenter diagnostic accuracy study, six centers	298 infants (832 examinations)	Median 29 (26–30) weeks b	Two scores compared: six anterolateral zones, and the same plus the posterior fields	Birth, DOL 3, 7, 14, 21 (no day-28 assessment)	msBPD	≥8 on DOL 7 (anterolateral score)	Day 7 AUC 95% CI 0.74–0.85; sensitivity 70%, specificity 79%; positive likelihood ratio 3.3, negative likelihood ratio 0.38	The authors characterize the accuracy as moderate; adding the posterior fields significantly improved accuracy only on day 14 (*p* = 0.01)
Raimondi et al., 2021 [48]	Prospective multicenter cohort	240 preterm neonates	Three prespecified strata: 25–27, 28–30, 31–33 weeks	Six zones defined by the midclavicular, anterior axillary and posterior axillary lines; 0–3 per zone; total 0–18	Birth, then weekly throughout admission	NR d	NR	Day 7 in infants 25–30 weeks: AUC 0.82 (0.71–0.93). Sensitivity and specificity NR	The score trajectory was gestational age-dependent and correlated with the SpO_2_/FiO_2_ ratio throughout admission
Li et al., 2023 [51]	Prospective observational cohort, single center	150 infants (63 with msBPD)	<32 weeks; mean 28.7 ± 1.6 weeks; BW 1176 ± 225 g	Six anterolateral zones; 0–3 per zone; total 0–18; posterior fields not scanned	DOL 3, 7, 14, 21	msBPD: support at 36 weeks PMA or at discharge. Secondary outcome: any BPD, NIH/NICHD 2001 [2]	msBPD, DOL 7: ≥5. Any BPD, DOL 14: ≥4	msBPD: AUC 0.78 (0.71–0.84), sensitivity 71.4%, specificity 73.6%. Any BPD: AUC 0.87 (0.81–0.92), sensitivity 72.9%, specificity 90.7%	Performance was gestational age-dependent: at 23–27 weeks the score added little beyond GA alone, and at ≥28 weeks a five-variable clinical model outperformed the score (AUC 0.91 versus 0.77)
De Luca et al., 2026 (PATH-BPD) [44]	Prospective multicenter cohort, three European centers	347 infants (80, 79 and 89 with msBPD according to the three definitions)	≤30 weeks	Quantitative LUS; anterolateral fields	DOL 10, 21, 28, and 34 and 36 weeks PMA (earliest assessment on day 10)	Three definitions applied in parallel: NIH/NICHD 2001 [2], NICHD 2018 [46] and Jensen 2019 [3]	No cutoff; trajectory analysis	Lung aeration evolution β(t) = 0.227 (0.152–0.302), *p* < 0.001; no absolute score values reported	Aeration and oxygenation were already poorer on day 10 in infants who subsequently developed msBPD, and the difference widened over time, peaking at 26 days; the trajectory was consistent across all three BPD definitions

**Table 4:** Meta-analyses of lung ultrasound for the prediction of bronchopulmonary dysplasia. Pooled estimates are shown alongside the time point to which they apply, to allow for direct comparison with the individual studies in Table 3. Abbreviations as in Table 3.

Meta-Analysis	Studies/Neonates	Time Point	AUC	Pooled Sensitivity	Pooled Specificity	Comment
Pezza et al., 2022 [32]	7/1027	Days 7–14	0.85–0.87	70–80%	80–87%	The classical six-area and the extended ten-area scores showed comparable accuracy, with no significant difference at any time point
Zhang et al., 2025 [33]	10/1274	DOL 7	Any BPD 0.87; msBPD 0.80	Any BPD 75%; msBPD 69%	Any BPD 83%; msBPD 79%	Day 7 gave the highest accuracy; performance was consistently lower when the outcome was restricted to msBPD
Han et al., 2025 [50]	22/2038	Days 1–3	0.81	75%	74%	Accuracy improved with postnatal age; the pooled estimates at days 14–21 exceed those reported by individual multicenter studies at day 7
		DOL 7	0.88	78%	83%	
		DOL 14	0.87	78%	84%	
		DOL 21	0.92	85%	86%	

## Data Availability

No new data were created or analyzed in this study. Data sharing is not applicable to this article.

## Abbreviations

The following abbreviations are used in this manuscript:

AI	Artificial Intelligence
AUC	Area Under the Receiver Operating Characteristic Curve
BPD	Bronchopulmonary Dysplasia
CPAP	Continuous Positive Airway Pressure
CNNs	Convolutional Neural Networks
CT	Computed Tomography
DOL	Day of Life
ELBW	Extremely Low Birth Weight
FiO_2_	Fraction of Inspired Oxygen
ICC	Intraclass Correlation Coefficient
LISA	Less Invasive Surfactant Administration
LUS	Lung Ultrasound
MAP	Mean Airway Pressure
MeSH	Medical Subject Headings
MIST	Minimally Invasive Surfactant Therapy
MRI	Magnetic Resonance Imaging
ms-BPD	Moderate-to-Severe Bronchopulmonary Dysplasia
NICHD	National Institute of Child Health and Human Development
NIH	National Institutes of Health
ROC	Receiver Operating Characteristic
SANRA	Scale for the Assessment of Narrative Review Articles
SpO_2_	Peripheral Oxygen Saturation
